# Digital finance and regional economic resilience: Evidence from 283 cities in China

**DOI:** 10.1016/j.heliyon.2023.e21086

**Published:** 2023-10-17

**Authors:** Shiying Hou, Yining Zhang, Liangrong Song

**Affiliations:** a Business School, University of Shanghai for Science and Technology, Shanghai, China; b Business School, Yancheng Teachers University, Jiangsu, China

**Keywords:** Digital finance, Regional economic resilience, Evaluation system, Influence mechanism, Threshold effect

## Abstract

Digital technology provided a new driver for the rapid recovery of the global economy in the post-COVID-19 era. This study examined how digital financing affected regional economic resilience. First, this study constructs a multidimensional regional economic resilience evaluation system and measures the economic resilience levels of 283 Chinese cities for 2012-2021–using the entropy value method. Then, panel data, mediation effect, and threshold effect models were constructed to empirically test the impact mechanism of digital finance (DF) on regional economic resilience. The results show that DF improves regional economic resilience, which is more evident in central and western cities. Capital allocation efficiency, regional innovation, and regional consumption are effective paths, whereas DF affects regional economic resilience by enhancing capital allocation efficiency, strengthening regional innovation capacity, and promoting resident consumption. It is worth noting that excessive financialization can mask the role of DF. These conclusions provide new evidence clarifying the role of DF in promoting rapid economic recovery in the post-COVID-19 era.

## 1 Introduction

A major challenge for countries is how to resume economic development in the post-COVID-19 era, and how to improve economic resilience. The economic resilience reflects an economy's ability to resist, recover, reorganize, and innovate [1,2]. When unforeseen events such as economic downturns, natural disasters, technological changes, and global market turmoil occur, a more resilient economy can effectively resist shocks, mitigate harmful consequences, and quickly return to a stable and sustainable development trajectory [3,4]. Existing research found that a diversified industrial structure, strong government, unified markets, efficient infrastructure, and mature division of labor are beneficial for economic resilience [[5], [6], [7], [8], [9]]. The complexity of economic resilience implies that its influencing factors are diverse [10]. As an essential part of the economic system, finance not only provides capital for enterprise production and guides residents' consumption and savings but is also a stable tool for the government to relieve downward pressure on the economy [11,12]. When accidents happen, adequate capital support can guarantee for economic recovery [13]. The resumption of work and production by enterprises, consumption by residents, and social stability all generate a strong demand for financial resources. Therefore, economic resilience from the perspective of financial functioning is an interesting topic.

With the integration of digital technology and traditional finance, the efficiency of traditional finance services has improved and a new service model of digital finance (DF) has been developed. Compared to the offline service model of traditional financial services, DF utilizes digital technologies such as artificial intelligence, blockchain, cloud computing, and big data to overcome the geographic limitations of financial services [14,15]. On the other hand, DF's new features of platformization, digitization, and inclusion improve the efficiency of information transfer and lower the threshold and cost of accessing financial services for different groups [[16], [17], [18]]. Efficient digital financial services provide new impetus for regional economic growth [19]. McKinsey's 2017 statistical report shows that DF is expected to increase the GDP in emerging economies by 6 % and create 95 million jobs by 2025.^1^ Especially during COVID-19, many economic activities were restricted, and the non-contact service model of DF broke through the time and regional restrictions on capital flows and met the actual needs of residents' consumption, corporate financing, and international trade [[20], [21], [22]]. Data from China's National Bureau of Statistics show that in 2020, under the strict control policies of COVID-19, China's economy still achieved stable growth, with a GDP growth rate of 2.2 %, of which the digital economy contributed 55 %.^2^ Clearly, there is a link between the digital economy and the Chinese economy's recovery. Thus, an interesting question is, what is the role of DF? Have the roles of financial stabilizers been fulfilled? This study examined the relationship between DF and economic resilience to provide new evidence of rapid economic recovery.

This study constructed an empirical model to test the impact of DF on regional economic resilience using data from Chinese cities. First, an evaluation system for regional economic resilience was constructed to measure the economic resilience index. Second, we examined whether DF can enhance the resilience of regional economies from the perspective of financial stabilizers. Third, the threshold effect of excessive financialization on DF is further examined. Finally, we propose policy implications for improving the resilience of regional economies.

The contributions of this study are as follows. First, we study the impact of DF on regional economic resilience from the perspective of financial innovation, which can more clearly identify the role of finance in economic resilience, helping improve the economy's response to economic shocks and recovery. Second, we analyze the impact mechanism of DF on regional economic resilience and examines the path through which DF affects regional economic resilience from three perspectives: capital allocation efficiency, technological innovation, and resident consumption. Third, a regional economic resilience indicator evaluation system is constructed based on three dimensions: resilience and recovery capacity, adaptation and adjustment capacity, and innovation and transformation capacity. They fully reflect the resilience of an economic system and improve the accuracy of measuring economic resilience.

The remainder of this paper is organized as follows. Section 2 provides a literature review. Section 3 presents the theory and hypotheses. Section 4 describes the study model design, variables, and data. Section 5 reports and discusses the results. Finally, we offer conclusions and policy implications.

## 2 Literature review

### 2.1 Economic resilience

In physics, resilience refers to the rate at which a system returns to its pre-disturbance state. Economic resilience is the self-recovery and adjustment function of an economy after exposure to external shocks [4]. Higher economic resilience can help economies quickly return to their previous growth paths or reallocate resources to expand new growth paths [23,24]. Existing research on regional economic resilience focuses on the measurement and influencing factors. Comprehensive evaluation systems and sensitivity factors are commonly used to measure economic resilience [25,26]. For example, a comprehensive evaluation system of economic resilience was constructed in terms of GDP, environment, resources, and technology, and was calculated using the weight division method [27]. However, the limitations of these indicators can lead to inaccurate results, and the embeddedness of the economic structure cannot be ignored [28]. Economic data based on counterfactuals can also reflect the sensitivity of regional economies to shocks [29]. For example, the difference between GDP growth and unemployment rates over time can represent the level of regional economic resilience [3,8]. However, a sensitivity test based on established facts cannot scientifically predict the future directions of economic resilience.

In terms of the factors influencing economic resilience, industrial structure, labor force, finance, trade, and government governance are the basic frameworks for analyzing regional economic resilience [4]. Scholars conducted research on aspects of economic agglomeration, industrial structure, social capital, policy and institutional environment, and regional culture [7,30,31]. The results show that the agglomeration of economic factors and the diversification of industrial structure are not only reflected in the “automatic stabilizer” function of dispersing risks and resisting shocks [32,33], but also in promoting technological innovation and helping cities make adaptive structural adjustments during the recovery period [34]. In addition, regions with excellent business environments have better economic resilience [3], and their advantages in terms of innovation capacity, labor quality, government policy support, and capital mobility are important factors that enhance regional economic resilience [8,35,36].

### 2.2 The impact of DF

In recent years, digital technology has become mainstream owing to the widespread application of the Internet. The combination of traditional finance and digital technology created a new financial model represented by DF [37]. DF, supported by digital technology, improves the problem of high-risk premiums and high operating costs owing to information asymmetry in traditional finance [19]. The research on the role of DF mainly focuses on financing constraints, technological innovation, residents' income and consumption, social employment, and green development [21,[38], [39], [40], [41]]. From the aspect of financing constraints, DF is characterized by wide coverage, strong sharing, high convenience, and low interest rates [42]. These factors not only provide abundant capital for the production, R&D, and sales of enterprises [43], but also improves the efficiency of capital allocation of enterprises, which is beneficial to the sustainable growth of the economy [44]. In terms of technological innovation, DF reduces the information asymmetry of the “financial sector-investor-firm,” which enables better matching of resources with the risk characteristics of firms' innovation projects, providing the necessary conditions for improving firms' technological innovation [45]. Regarding residents' income and consumption, DF allows more people to participate in the financial market. On the one hand, convenient payment methods satisfy residents' differentiated consumption needs [46]. By contrast, low-threshold financial services increase residents' access to credit resources. From the social development perspective, DF can effectively solve the social problems of employment, poverty, and energy consumption [47]; however, the digital divide can exacerbate social inequality. Scholars also studied the relationship between DF and economic resilience, demonstrating that digitalization and DF enhance economic resilience through significant positive spatial spillover effects [7]. Among these, the role of digital inclusive finance is more obvious in regions with favorable financial markets and business environments [3]. However, some scholars proposed that the role of digitalization in economic resilience is heterogeneous and has an inverted U-shaped relationship [48].

Research on economic resilience and DF provides the basis for this study; however, there are still research gaps. First, financing is important for economic growth. However, the impact of financial innovation on economic resilience has been neglected. Although some scholars concentrated on the relationship between DF and economic resilience, there are large differences in research results [3,7,48]. Simultaneously, studies based on spatial effects, financial market environment, and business environment do not show the complete mechanism by which DF affects economic resilience. There is a gap in the understanding of the impact of DF on economic resilience. Second, economic resilience reflects an economy's multidimensional ability to cope with shocks. The measurement of single-dimensional indicators centered on GDP and the unemployment rate cannot fully reflect the level of resilience of an economic system [27,29]. It is necessary to construct a sustainable comprehensive evaluation system that includes the abilities of resistance, resilience, adaptability, and innovation in accordance with the characteristics of economic resilience. Therefore, this study examines the influence of DF on regional economic resilience and analyses its path mechanism, which may provide new insights into the rapid recovery of the global economy under the impact of COVID-19.

## 3 Theoretical mechanism and research hypothesis

The advantages of DF are reflected in its high-efficiency information transfer and resource allocation. When economic growth suffers from shocks, low-cost information transactions provided by digital technology tools can provide efficient financial services for the economy to respond to risks [49]. This can overcome the dilemma of corporate financing constraints and maintain corporate production [50]. In addition, financial discrimination makes it difficult for many groups to obtain loans. DF is important and beneficial for addressing financial exclusion, helping discriminated groups obtain financial resources, and maintaining the sustainability of production and consumption [51]. With improved financial efficiency, financial institutions can also respond quickly to the impact of economic fluctuations on business production and household consumption, which has a positive impact on improving the ability of the urban economic system to withstand and absorb shocks [46]. Second, the speed of recovery of an urban economic system after a shock is closely related to a city's ability to integrate internal resources and adjust its structure to adapt to a new external environment [49]. DF created financial products and services through digital platforms, which effectively break geographical restrictions so that financial services are no longer limited to real life. Internet platforms can provide financial services to enterprises and residents more quickly, enhance the liquidity of funds in the market, and improve the efficiency of financial resource allocation [52]. In addition, DF provides financial services based on big data analysis. Dynamic data analysis can clarify trends in changes in the external economic environment over time, which also improves the adaptability of regional economies [53]. Hypothesis 1 is as follows:

Hypothesis 1 DF has a positive effect on the regional economic resilience.

Companies encounter financing constraints under the impact of changes in internal and external environments. Effective capital allocation can help enterprises stably implement production and operation plans, which are also key to achieving sustainable development of a regional economy [54]. DF can effectively reduce financial frictions. The digital online platform expands the scale of finance, reduces financial transaction costs, breaks regional financial market segmentation, reduces the difference in loan cost caused by market barriers, and improves the efficiency of regional capital allocation [49,50]. In addition, compared with traditional finance, DF relies on big data and artificial intelligence to improve the accuracy of financial asset pricing, expand the sources of corporate financing, and positively affect corporate financing, risk management, payment, and investment decision-making [9]. This is conducive to reducing the risk of capital misallocation and improving the capital allocation efficiency [55]. Improving the efficiency of regional fund allocation will accelerate the flow of funds in the market, provide support for stable investment and production, increase profitability, and strengthen enterprises’ ability to withstand shocks. Hypothesis 2 is as follows:

Hypothesis 2 DF affects regional economic resilience by improving capital allocation efficiency.

Technology is the core driving force of economic growth [9]. Enterprises rely on advanced technologies to gain an advantage in terms of market competition. Particularly under the impact of an economic crisis, the irreplaceability of core technologies will still benefit the region and become the dominant force guiding economic recovery. First, DF helps to compensate for the shortcomings of traditional financial services in technological innovation. DF offers the advantages of wide coverage, large-scale funding, and low financing costs. This can effectively lower the threshold of financial services and expand their boundaries of financial services [38], providing more efficient financial services for enterprises’ technological innovation [50]. Second, the application of digital technology provides DF with a low-cost advantage for information acquisition [42]. This can effectively alleviate information friction between the market and enterprises, and guide more innovative resources in technological innovation [53]. As the scale of the technology trading market expands, DF exhibits significant scale effects, reducing the cost of innovation and increasing marginal revenue [56]. Finally, DF improves the financial market environment, which positively affects competition and cooperation among regional innovation entities. The economies in regions with high levels of innovation will gain a greater ability to adapt to external shocks [57]. The benefits of new technologies can also become a new way to enhance the resilience of the regional economy.

Hypothesis 3 DF affects regional economic resilience by improving regional innovation.

Under the pressure of an economic downturn, the role of investment and exports in stimulating economic growth is suppressed. To drive economic growth, it is more advantageous to rely on consumption and domestic demand. According to income theory, income is the main factor determining household consumption; however, household consumption is also affected by budget and personal wishes. DF lowered the threshold for financial services and changed the lending model. It allows financial institutions to provide low-cost, low-threshold loans to consumers that can supplement the existing income of the population and encourage further consumption [9]. In addition, DF provides a new consumption model. Internet consumption platforms, represented by third-party payments and e-commerce, provide convenience for residents' payments [18], which has a positive effect on improving household consumption experience and stimulating residents' willingness to consume [40]. By strengthening the role of consumption in economic growth, enterprises’ enthusiasm for production will also increase. Eventually, a cycle of consumption and production forms in the economic system, which can improve the resilience of the regional economy and provide space for regional economic recovery.

Hypothesis 4 DF affects regional economic resilience by improving the residents’ level of consumption.

## 4 Research design

### 4.1 Model

To clarify the direct impact and influence path of DF on regional economic resilience, we used regional economic resilience and DF as explanatory variables and selected control variables to construct a panel data model of DF and regional economic resilience.

(1) $$RER_{i,t}=\alpha_{0}+\alpha_{1}DF_{i,t}+\alpha_{2}CV_{i,t}+\lambda_{i}+\mu_{t}+\varepsilon_{i,t}$$

in Equation (1), *RER* represents the regional economic resilience, *DF* represents DF, and *CV* is other control variables that affect the regional economic resilience. *i* represents the area, *t* represents the time, *λ* represents the individual effect, *μ* represents the time effect, and ε is the random error term.

To test Hypotheses 2, 3, and 4, we constructed a mediating effect model to analyze the path by which DF influences regional economic resilience. The mediating effect model was primarily used to study the influence of the explanatory variable on the explanatory variable through the mediating variable. Equations (2), (3) test the mediation effect.

(2) $$M_{i,t}=\beta_{0}+\beta_{1}DF_{i,t}+\beta_{2}CV_{i,t}+\lambda_{i}+\mu_{t}+\varepsilon_{i,t}$$

(3) $$RER_{i,t}=\gamma_{0}+\gamma_{1}DF_{i,t}+\gamma_{2}M_{i,t}+\gamma_{3}CV_{i,t}+\lambda_{i}+\mu_{t}+\varepsilon_{i,t}$$

In Equations (2), (3), *M* is the mediating variable. First, the significance of $\alpha_{1}$ is the basis for testing its mediating effect. When $\beta_{1}$, $\gamma_{1}$, $\gamma_{2}$ pass the significance test, and $\gamma_{1}$ <$\alpha_{1}$, we find a mediating effect, with *M* as the mediating variable. If $\beta_{1}$, $\gamma_{1}$, or $\gamma_{2}$ fails the significance test, then a mediating effect may not exist, or we need to further pass the Sobel test to make a judgment.

### 4.2 Variables

(1) Regional economic Resilience (*RER*)

Regional economic resilience is the explanatory variable. It is necessary to establish a multidimensional indicator evaluation system to scientifically evaluate regional economic resilience. We established a comprehensive index system of regional economic resilience based on three dimensions: resistance and recovery capabilities, adaptation and adjustment capabilities, and innovation and transformation capabilities [58]. Resistance and recovery are the abilities of an economy to withstand and recover from shocks and are closely related to the previous economic foundation [4,[59], [60], [61], [62]]. Adaptation and adjustment are internal changes in an economy after a shock, including self-adaptability and factor allocation [44,63,64]. Transformation and innovation are the abilities of an economy to create new development paths after a shock, including technological progress and economic structure optimization [56,65,66]. Table 1 presents the regional economic resilience evaluation system used in this study.

To calculate regional economic resilience, we used the entropy method to calculate the weights based on the index evaluation system in Table 1. Entropy is a mathematical method used to evaluate the degree of dispersion of an index. This can be used to determine the influence of the indicators. Because the measurement units of various indicators are not uniform, they must be standardized. Because of the different meanings of the positive and negative indicators, we used different equations to calculate them positive and negative indicators, respectively, as follows.

(4) $$Z_{ij}=\left(x_{ij}-\min\;x_{j}\right)/\left(\max\;x_{j}-\min\;x_{j}\right)$$

(5) $$Z_{ij}=\left(\max\;x_{j}-x_{ij}\right)/\left(\max\;x_{j}-\min\;x_{j}\right)$$

in Equations (4) and (5), $Z_{ij}$ is the standardized index value, $x_{ij}$ is the value of the *i*-th level *j* index, $\max\;x_{j}$ is the maximum value, and $\min\;x_{j}$ is the minimum value. Equations (4) and (5) were used to calculate the positive and negative indicators, respectively. Equation (6) is used to calculate the weight of the index, and $d_{j}$ is the coefficient of variation.

(6) $$w_{j}=d_{j}/\sum_{j=1}^{n}d_{j}$$

We used the weighted average method to measure regional economic resilience, as shown in Equation (7).

(7) $$RER=\sum_{j=1}^{n}w_{j}*Z_{ij}$$

**Fig. 1:**
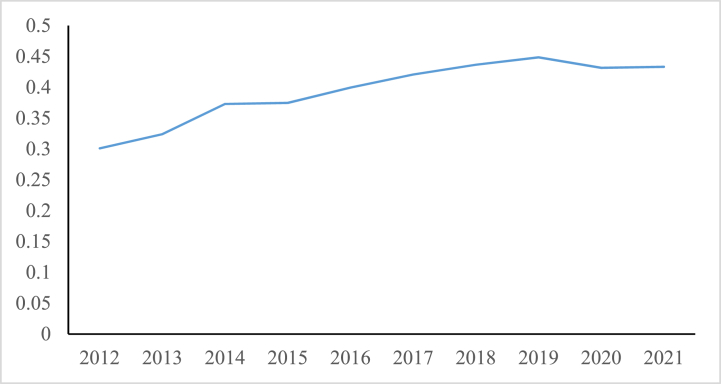
The average value of economic resilience of 283 cities in China from 2012 to 2021.

Fig. 1 illustrates the average economic resilience values.

(2) Digital finance (DF)

DF was the core explanatory variable in this study. We use the digital financial index calculated by the Internet Finance Research Center of Peking University, which measures the three dimensions of coverage, utilization, and digital support services. These include: digital account size, digital payment, digital investment, digital credit, digital insurance, digital credit collection, and service efficiency. The coverage is a measure of the number of people covered by DF. The utilization rate is the frequency of the actual use of digital financial services in the region. Service efficiency is the convenience and efficiency of DF in the region.

(3) Other variables

According to the theoretical analysis, we used the efficiency of capital allocation (*rca*), regional innovation level (*inv*), and regional consumption level (*consum*) to test the mediating effect of DF on regional economic resilience. We used the elasticity coefficient of capital as the output value to measure the efficiency of capital allocation in the region [67]. In Equation (8), *I* is the total fixed capital of the region, G is the GDP of the region, *i* is the area, *t* is the time, and *c* is the intercept term. η is the elastic coefficient of capital to economic output used to measure *rca*. Regional innovation reflects urban innovation output. We use the number of patent applications per 10,000 people to represent *inv* [56]. The regional consumption level (*consum*) is expressed as the total consumption by regional households. To eliminate the influence of population size, we use the per capita consumption expenditure of regional urban households.

(8) $$\ln\left(\frac{I_{i,t}}{I_{i,t-1}}\right)=c_{0}+\eta_{i,t}\;\ln\left(\frac{G_{i,t}}{G_{i,t-1}}\right)+\varepsilon_{i,t}$$

(4) Control variables

To avoid the interference of other variables on the estimation results, we chose other control variables by referring to relevant literature. Industrial structure (*ind*) is the ratio of the output value of the tertiary industry to the output value of the secondary industry to measure [68]. We include the level of human capital (*human*) as a high level of human capital reflects the skills and production potential of the regional labor force, which is very important for the development of the regional economy [69]. We used the number of college students per 10,000 people in each city to represent the level of human capital. Level (*ins*). Transportation occupies an important position in the regional economy and trade [70]. We selected the urban highway mileage to measure the level of regional infrastructure construction. Urbanization rate (*urb*). Economic growth requires the support of urban areas. The proportion of the urban population represents urbanization.

### 4.3 Data

We selected 283 prefecture-level cities in China from 2012 to 2021 because the levels of DF and economic growth capacity in China provide valid evidence. All economic data for this study were obtained from the CSMAR database, National Bureau of Statistics of China, and China Regional Yearbook. To reduce the error caused by extreme values, we winsorize 1 % and 99 % of the data samples. To test for multicollinearity, we used the variance inflation factor (VIF) to test the benchmark model. The test results show that the variance inflation factor (VIF) value is less than 3, rejecting the multicollinearity assumption. Table 1 presents the descriptive statistics.

**Table 1:** Descriptive statistics of the variables.

Variable	Unit	Obs	Mean	Std. Dev.	Min	Max
*RER*	1	2830	0.403	0.256	0.027	2.618
*DF*	1	2830	119.542	46.838	16.220	334.48
*cae*	1	2830	0.571	0.471	0.117	4.835
*inv*	1	2830	9.843	25.182	0	341.724
*consume*	RMB (yuan)	2830	22038	6331.546	6720	48272.000
*ind*	1	2830	1.180	0.657	0.518	5.167
*human*	1	2830	9.174	16.483	0.010	129.25
*urb*	1	2830	0.566	0.124	0.368	0.893
*ins*	Kilometer	2830	13226.98	10237.22	614	174284

## 5 Results and discussion

### 5.1 Regression results

Equation (1) was used to test the baseline relationships between the variables. Because the samples were panel data, we used the Hausman test (Prob > chi2 = 0.0001) for fixed and random effects. Clearly, the fixed-effects model was more appropriate. Based on the comparability of the results, we present the results of the ordinary least squares (OLS) model with and without control variables and the fixed-effects model (FE). As shown in Table 2, the coefficients of DF were 0.224 and 0.229 for OLS and FE, respectively, and were significant. This indicates that DF can enhance regional economic resilience. In addition, if we consider the role of the control variables, DF also promotes regional economic resilience. A comparison of the results further validates Hypothesis 1. DF has a positive effect on regional economic resilience. Our results also show that industrial structure, human capital, urbanization, and infrastructure contribute to regional economic resilience. This is because the optimization of the industrial structure, higher education levels, improvement of urbanization, and improvement of basic transportation conditions are all key factors affecting the stability of a regional economy. This is consistent with the reality of China's economy.

**Table 2:** Benchmark regression model results.

Variable	(1) OLS	(2) OLS	(3) FE	(4) FE
	Dependent variable = *RER*			
*DF*	0.224***	0.210***	0.229***	0.218***
	(5.77)	(5.28)	(5.50)	(5.77)
*ind*		0.373**		0.374**
		(2.24)		(2.25)
*human*		0.138**		0.138**
		(2.32)		(2.32)
*urb*		0.106		0.107*
		(1.59)		(1.70)
*ins*		0.049***		0.051***
		(5.54)		(5.56)
*constant*	−0.432**	−0.370**	−0.457**	−0.373**
	(-2.18)	(-2.07)	(-2.15)	(-2.09)
Year			YES	YES
City			YES	YES
Observation	2830	2830	2830	2830
*R*^2^	0.187	0.223	0.195	0.229

Note: ***, **, and * represent p-values of 1, 5 %, and 10 %, respectively.

### 5.2 Heterogeneity test

China's economic development has considerable regional differences. For example, eastern cities have significant advantages over central and western cities in terms of economic scale, digital infrastructure development, and marketization levels. This study examines the heterogeneous impact of DF on the economic resilience of cities in different regions. In accordance with the National Bureau of Statistics of China (NBS)'s criteria for city location, we divide the 283 sample cities into three groups for empirical testing: eastern, central, and western. In the results in Table 3, the DF coefficients of the cities in the east, center, and west are 0.172, 0.241, and 0.328, respectively, and all pass the significance test. This finding indicates that the positive effect of DF on economic resilience still exists under the influence of regional differences. We should note that DF has a greater marginal effect on central and western cities than on eastern cities.

**Table 3:** Regional heterogeneity test results.

Variable	(1) Eastern	(2) Central	(3) Western
*DF*	0.172**	0.241***	0.328**
	(2.16)	(5.09)	(5.13)
*Ind*	0.429***	0.460**	0.257*
	(3.33)	(2.31)	(1.69)
*Human*	0.140**	0.127**	0.131**
	(2.29)	(2.24)	(2.26)
*Urb*	0.118**	0.133*	0.099*
	(2.04)	(1.73)	(1.70)
*Ins*	0.088***	0.104***	0.136***
	(5.77)	(5.78)	(5.82)
*Constant*	−0.469**	−0.420**	−0.411**
	(-2.11)	(-2.08)	(-2.07)
*Year*	YES	YES	YES
*City*	YES	YES	YES
*Observation*	1200	800	830
*R*^*2*^	0.253	0.249	0.231

Note: ***, **, and * represent p-values of 1, 5 %, and 10 %, respectively.

### 5.3 Robustness test

To ensure the robustness of the results, we conducted a series of tests using instrumental variables, replacing the explanatory model with core variables. Table 4 summarizes the results.

**Table 4:** Robustness test results.

Variable	(1) First stage	(2) Second stage	(3) GMM	(4) Substitution variable
	Dependent variable = *df*	*RER*	*RER*	*RER_1*
*tel*	0.742**			
	(2.03)			
*DF*		0.197***		0.527**
		(4.35)		(2.42)
*DF*_*t-1*_			0.201**	
			(2.17)	
*ind*	0.421**	0.262*	0.316**	0.469**
	(2.18)	(1.72)	(2.02)	(2.20)
*human*	0.064	0.114**	0.126*	0.226**
	(1.47)	(2.10)	(1.80)	(2.33)
*urb*	0.163**	0.098	0.114	0.142**
	(2.23)	(1.43)	(1.58)	(2.17)
*ins*	0.281***	0.049***	0.033***	0.140***
	(4.07)	(5.54)	(3.95)	(5.62)
*constant*	−1.053***	−0.382**	−0.427**	−0.407**
	(-2.72)	(-2.11)	(-2.28)	(-2.16)
*Year*	YES	YES	YES	YES
*City*	YES	YES	YES	YES
*F statistic*	1102.98			
*C-D Wald F statistic*		1248.58		
*K–P rk LM statistic*		1875.39		
*Observation*	2830	2830	2830	2830
*R*^*2*^	0.187	0.223	0.219	0.207

Note: ***, **, and * represent p-values of 1, 5 %, and 10 %, respectively.

First, finance is a component of the economy and reverse causality may lead to endogeneity problems, which we test using instrumental variables. We use the number of urban landline telephones in 1984 as an instrumental variable [61]. DF is correlated with Internet use, and telephones are consistent with Internet penetration. Simultaneously, the number of fixed phones has a relatively small impact on current economic growth. This meets the requirement for IV exclusivity of the instrumental variable. In Column 2 of Table 4, the Kleibergen-Paap rk LM statistic is 1875.39, and the C-D Wald F statistic is 1248.28. They reject the hypotheses of insufficient instrumental variable identification and weak instrumental variables. Thus, our chosen instrumental variables are effective. Simultaneously, the coefficient of the DF variable in Column (2) is 0.197, which is consistent with the benchmark regression results. These findings indicate that DF plays a significant role in economic resilience.

Secondly, the generalized matrix model (GMM) can be effective in reducing the error of endogeneity on statistical results. In this study, the benchmark results are also retested using the GMM, and the results in Column 3 show that the coefficient of the DF lag term is 0.201, which indicates that the effect of DF still exists after replacing the benchmark model.

Finally, to avoid errors caused by variable measurements, we substituted the calculations of explanatory variables for robustness testing. Based on the rate of change in GDP [3], we calculated the regional economic elasticity (RER_1) as in Equation (9). $\Delta G_{it}$ is the actual GDP growth rate of region *i* in period *t*, and $\Delta E_{it}$ is the average national GDP growth rate in period *t*. A larger value indicates a more resilient regional economy. Column (4) presents the regression results obtained by varying the RER calculation method. The coefficient of DF was also significantly positive.

(9) $$RER\_1_{it}=\frac{\Delta G_{it}-\Delta E_{it}}{\Delta E_{it}}$$

### 5.4 Transmission mechanism

Based on the assumptions of mechanism analysis, we validated the paths of the mediating variables. In Table 5, Columns (1) and (4) show the test results for the mediating effect of *cae*. The coefficient of DF is 0.859, indicating that it can improve the capital allocation efficiency. In Column (4), the coefficients of DF and *cae* are 0.173 and 0.052, respectively. Combined with the results of the mediation model, we find a partial mediation effect and DF affects regional economic resilience by improving capital allocation efficiency. Thus, Hypothesis 2 was confirmed. Columns (2) and (5) show the path tests for regional innovation levels. The coefficient of DF is significantly greater than 0, indicating that DF improves regional innovation levels. The effects of DF and the regional innovation level on regional economic resilience were 0.166 and 0.045, respectively. Hence, the regional innovation level also has a mediating effect. Thus, Hypothesis 3 was confirmed. In Columns (3) and (6), the coefficients of DF and *consum* are both greater than 0 and passed the significance level tests of 5 % and 1 %, respectively, indicating that the mediating path of regional consumption also exists. Thus, Hypothesis 4 was confirmed. Overall, capital allocation efficiency, regional innovation level, and residents’ consumption levels are effective paths by which DF affects regional economic resilience. Utilizing the functional advantages of DF to improve the efficiency of capital allocation, strengthen regional innovation capacity, and promote the growth of resident consumption can effectively increase regional economic resilience.

**Table 5:** Regression results: Mediating effect.

Variable	(1)	(2)	(3)	(4)	(5)	(6)
	*Cae*	*inv*	*consume*	*RER*	*RER*	*RER*
*DF*	0.859**	0.761**	1.012***	0.173***	0.166***	0.156***
	(2.28)	(2.15)	(3.09)	(5.31)	(5.26)	(5.29)
*cae*				0.052**		
				(2.07)		
*inv*					0.045*	
					(1.73)	
*consume*						0.061**
						(2.47)
*Controls*	YES	YES	YES	YES	YES	YES
*Year*	YES	YES	YES	YES	YES	YES
*City*	YES	YES	YES	YES	YES	YES
*Observation*	2830	2830	2830	2830	2830	2830
*R*^*2*^	0.427	0.362	0.493	0.240	0.238	0.242

Note: ***, **, and * represent p-values of 1 %, 5 %, and 10 %, respectively.

### 5.5 Reflections on the excesses of financialization

The rapid development of the financial sector and its prominence in industry are characteristics of modern developed market economies. The power DF brings to the financial sector could lead to a significant increase in the financial size of the economy. Financial stability theory explains the positive effect of finance on economic growth, but excessive financialization can also increase economic risk. The 2008 financial crisis is a profound example of this. We must consider the potential for DF to lead to excessive financialization. This study further tests the effect of excessive financialization on the role of DF using the threshold model [71,72] in Equation (10). Excessive financialization (*EF*) is the threshold variable, which is expressed as the ratio of the regional financial sector value-added to GDP. First, we determined the number of thresholds using 500 self-samples (bootstrap), and the results show the existence of a single threshold with an estimated threshold value of 8.21 % and the value is in the 95 % threshold interval, indicating a structural change in the relationship between finance and economic growth on both sides of the threshold. In Table 6, the coefficient of DF is 0.262 and is significant when the share of value-added financing in GDP is less than or equal to 8.21 %, indicating that DF can enhance regional economic resilience. When the value-added of the financial sector is greater than 8.21 % of GDP, the coefficient of DF is less than 0. This result indicates that excessive financialization increases regional economic risk. Excessive financialization can obscure the role of DF, and reasonable controls on the size of the financial sector are required to improve economic resilience.

(10) $$RER_{i,t}=\omega_{0}+\omega_{1}DF_{i,t}I\left(EF\leq \gamma \right)+\omega_{2}DF_{i,t}I\left(\gamma <EF\right)+\omega_{3}controls_{i,t}+\lambda_{i}+\eta_{t}+\varepsilon_{it}$$

**Table 6:** Regression results: Threshold effect.

Variable	(1)	(2)
*DF(EF* ≤ *8.21 %)*	0.262**	
	(2.33)	
*DF(EF*＞*8.21 %)*		−0.083*
		(-1.85)
*Controls*	YES	YES
*Year*	YES	YES
*City*	YES	YES

Note: ***, **, and * represent p-values of 1 %, 5 %, and 10 %, respectively.

### 5.6 Discussion

Undoubtedly, enhancing economic resilience is important for the rapid recovery of the regional economy in the post-COVID-19 era. This study differs from research on the role of industrial structure, transportation facilities, market environment, and government support on economic resilience [7,9,35] and examines the role of DF on regional economic resilience from the perspective of financial innovation, which enriches the research on finance and economic sustainability in the digital economy. The results show that DF can effectively enhance regional economic resilience, and this effect is more evident in central and western cities. In contrast to existing studies that focus only on the sensitivity of DF to changes in the GDP growth and unemployment rates [3,8], this study's comprehensive evaluation system of economic resilience based on resistance, resilience, adaptability, and innovation ability more scientifically embodies the reality of economic resilience. On this basis, the study of the role of DF is also more in line with the real need for sustainable development of the regional economy under external shocks. At the same time, capital allocation efficiency, regional innovation level, and the level of residents' consumption are the core paths through which DF influences regional economic resilience. Compared to a study of the external adjustment effect based on the financial market environment, business environment, and spatial spillover [7], this study scientifically confirms the internal path of DF affecting regional economic resilience using the mediation effect model. Finally, a study based on the threshold effect of excessive financialization shows that there is a stage difference in the role of DF on regional economic resilience, which is similar to the inverted U-shaped relationship [48]; however, this study explains the reason for this phenomenon from the perspective of excessive financialization. This is not only instructive for the scientific role of DF but also reflects the possible negative impacts of DF.

## 6 Conclusions and policy implications

The relationship between finance and the economy is a classic topic, and we re-examined the impact of DF on regional economic resilience from a resilience perspective. We constructed a multidimensional regional economic toughness evaluation system and measured economic toughness levels using data from 283 Chinese cities from to 2012–2021. Subsequently, we constructed panel data, a mediation effect, and a threshold effect model to test the mechanism of the impact of DF on regional economic resilience. The results show that DF can improve regional economic resilience and has a greater effect on cities in the central and western regions than on those in the east. The results of the mechanism test demonstrate that DF affects the resilience of regional economies by improving the efficiency of capital allocation, increasing the level of regional innovation, and promoting regional consumption. The results of the threshold effect test show that excessive financialization weakens the role of DF, and reasonable control of the regional financial share can make the economy more resilient.

Based on the research results, we propose several policy implications.

First, it is important to pay attention to the functions of financial stabilizers and take advantage of digital technology to release the role of finance. Therefore, the government should formulate a scientific strategy for the development of DF, promote the digital reform of the financial system, improve the digital financial service system and digital infrastructure, and enhance the risk-resistant capacity of regional economies. Simultaneously, financial institutions and enterprises should actively implement digital transformation strategies, reduce the threshold and cost of financial services, meet the diverse needs of more economic agents, and provide sufficient capital support for the rapid recovery of the economy.

Second, it is important to emphasize the positive role of the digital economy in capital allocation, technological innovation, and consumer consumption. On the one hand, utilizing digital technology to promote innovation in financial products and services and giving full play to the function of digital financial platformization enhances the liquidity of capital in the financial market, thus enhancing the efficiency of capital allocation. On the other hand, DF features low cost, high precision, and convenience in enterprise financing and residents' credit; provides sufficient capital support for regional innovation and residents' consumption; and strengthens the ability of the regional economy to adapt to adjustment and innovation transformation.

Finally, it is necessary to prevent the tendency toward excessive financialization. On the one hand, it encourages digital financial innovation so that financial resources can match the development needs of the real economy. On the other hand, it can strengthen the standardization, science, and normalization of financial supervision to correct the problem of excessive financialization and establish a barrier against internal and external systemic financial risks.

The limitations of this study are as follows. A multidimensional economic resilience evaluation system was constructed; however, the connotation of economic resilience was also enriched, and the selection of economic resilience sub-indicators should be adjusted dynamically. In addition, this study examined the relationship between DF and the economic resilience of cities from a macro perspective; financial institutions and fintech enterprises, as providers of digital financial services, better reflect the reality of the role of DF. Exploring the role of DF in the economic resilience of microeconomies is a worthwhile research direction.

## Data availability statement

Data will be made available on request.

**Table 1:** Regional Economic Resilience Evaluation System

Target	System	Indicator	References
Regional economic resilience	Resistance and recovery	Regional GDP per capita (yuan)	[4,[59], [60], [61], [62]]
		Disposable income per capita (yuan)	
		Resident savings balance (yuan)	
		Unemployment rate (%)	
		Foreign trade dependence (%)	
		Social security expenditure per capita (yuan)	
		Industrial concentration (%)	
		CPI (%)	
		GDP growth rate (%) Industrial waste emissions Population size	
	Adaptation and adjustment	Regional fixed investment (yuan)	[44,63,64]
		Total Retail Sales of Consumer Goods (yuan)	
		Employment rate (%)	
		Fiscal self-sufficiency rate (%)	
		Financial institution loan-to-deposit ratio (%) Environmental governance investment (yuan)	
	Innovation and transformation	Fiscal technology expenditure (yuan)	[56,65,66]
		R & D personnel ratio (%)	
		Number of patents granted (Includes green patents)	
		Sales revenue of new products (yuan)	
		Proportion of tertiary industry (%)	

## CRediT authorship contribution statement

**Shiying Hou:** Conceptualization, Data curation, Formal analysis, Investigation, Methodology, Writing – original draft, Writing – review & editing, Funding acquisition. **Yining Zhang:** Data curation, Investigation, Methodology, Writing – original draft. **Liangrong Song:** Conceptualization, Data curation, Investigation, Methodology, Project administration, Supervision, Writing – review & editing, Funding acquisition, Software.

## Funding statement

This research was supported by the Postdoctoral Science Foundation of China (2022M722147) and 10.13039/501100001809National Natural Science Foundation of China (71871144).

## Declaration of competing interest

The authors declare that they have no known competing financial interests or personal relationships that could have appeared to influence the work reported in this paper.

## Footnotes

1 Source: https://www.mckinsey.com/mgi/overview/in-the-news/what-digital-finance-means-for-emerging-economies.

2 Source: https://data.stats.gov.cn/easyquery.htm?cn=C01.
